# Tourniquets can further reduce perioperative blood loss in patients on dexamethasone and tranexamic acid during cemented total knee arthritis: a single-center, double-blind, randomized controlled trial

**DOI:** 10.1186/s10195-023-00698-3

**Published:** 2023-04-29

**Authors:** Wenyu Jiang, Xing Wang, Hong Xu, Menghan Liu, Jinwei Xie, Qiang Huang, Ronghua Zhou, Zongke Zhou, Fuxing Pei

**Affiliations:** 1 Department of Orthopedics, Orthopedic Research Institute, West China Hospital, Sichuan University, No.37, Guoxue Road, Wuhou District, Chengdu, 610041 Sichuan People’s Republic of China; 2 Department of Anesthesiology, West China Hospital, Sichuan University, No.37, Guoxue Road, Wuhou District, Chengdu, 610041 Sichuan People’s Republic of China; 3 Department of Orthopaedics, The Third People’s Hospital of Chengdu, Southwest Jiao Tong University, No.37, Guoxue Road, Wuhou District, Chengdu, 610041 Sichuan People’s Republic of China

**Keywords:** Total knee arthroplasty, Tourniquet, Dexamethasone, Tranexamic acid, Blood loss

## Abstract

**Background:**

Multiple doses of dexamethasone and tranexamic acid can inhibit postoperative inflammation and reduce fibrinolysis and perioperative blood loss in total knee arthroplasty. In this single-center, double-blind, randomized clinical trial, the aim was to investigate whether applying a tourniquet to patients on dexamethasone and tranexamic acid could further reduce perioperative blood loss.

**Materials and methods:**

Patients who underwent cemented total knee arthroplasty at our hospital were randomized to receive a tourniquet (*n* = 71) or not (*n* = 70) during the procedure. All patients received multiple doses of dexamethasone and tranexamic acid perioperatively. The primary outcome was perioperative blood loss, while secondary outcomes were surgery duration, postoperative laboratory indices of inflammation and fibrinolysis, range of knee motion, VAS pain score, knee circumference, knee swelling rate, homologous transfusion, albumin use, and complications.

**Results:**

Using a tourniquet was associated with significantly lower intraoperative blood loss (*P* < 0.001) and total blood loss (*P* = 0.007) as well as significantly shorter surgery duration (*P* < 0.001). In contrast, the tourniquet did not significantly affect hidden blood loss, postoperative inflammation or fibrinolysis, range of knee motion, VAS pain score, knee circumference, knee swelling rate, homologous transfusion, albumin use, or complications.

**Conclusions:**

The results of this randomized clinical trial demonstrate that applying a tourniquet during cemented total knee arthroplasty to patients receiving multiple doses of dexamethasone and tranexamic acid can further reduce perioperative blood loss without increasing the risk of inflammation, fibrinolysis, or other complications. Thus, it is advised to use tourniquets combined with dexamethasone and tranexamic acid to reduce perioperative blood loss and avoid tourniquet-related adverse events.

*Level of evidence:* Therapeutic Level I.

*Trial registration* Chinese Clinical Trail Registry, ChiCTR2200060567. Registered 5 June 2022—retrospectively registered, http://www.chictr.org.cn/showproj.aspx?proj=171291.

## Introduction

Total knee arthroplasty (TKA) is considered the most effective therapy for patients with end-stage knee arthritis because it can relieve pain and improve joint function [1]. However, TKA is usually accompanied by massive intraoperative bleeding and a poor surgical field of view, which may prolong surgery and inhibit cement penetration [2, 3]. Longer surgery increases the risk of surgical site infection, re-operation, and blood transfusion [4].

Applying tourniquets during surgery can maintain a clear surgical field by reducing intraoperative blood loss, thereby shortening the operation time. However, their use in orthopedic procedures such as TKA is controversial, given that they can increase the risk of postoperative thigh pain, limb swelling, nerve palsy, muscle injury, and deep vein thrombosis [2, 5, 6]. Tourniquets can also increase hidden blood loss, resulting in greater total blood loss [2, 5, 7].

We wondered whether the disadvantages of tourniquets would be mitigated by the perioperative dexamethasone typically used during TKA to inhibit postoperative inflammation, vomiting, and thigh pain [8–11]. In particular, multiple doses of dexamethasone can enhance postoperative recovery [9, 11, 12]. In addition to dexamethasone, TKA patients are often given tranexamic acid, which inhibits plasminogen activation to prevent fibrinolysis, thereby reducing hidden blood loss [13–17]. Numerous studies have shown that intravenous perioperative tranexamic acid can reduce postoperative blood loss and transfusion rates without increasing the risk of deep vein thrombosis or pulmonary embolism [18–25].

Here we tested the safety and efficacy of applying tourniquets to patients undergoing cemented TKA involving multiple doses of dexamethasone and tranexamic acid.

## Materials and methods

### Patients and randomization

This study has been reported in line with Consolidated Standards of Reporting Trials (CONSORT 2010) Guidelines. This single-center, double-blind, randomized controlled trial was approved by the Biomedical Ethics Committee of Sichuan University West China Hospital (date February 8, 2022/no. 2021–1699). All patients who were candidates for cemented TKA at our hospital from February 2022 to June 2022 were considered for inclusion. Patients were included if they underwent TKA for knee osteoarthritis in our hospital and showed a flexion-contracture deformity  of < 20°, varus or valgus deformity of < 20° [26]. Each patient provided written informed consent before surgery.

Patients were excluded if they had a history of knee infection, had a level of hemoglobin of < 100 g/L or coagulopathy, were using anticoagulants or antiplatelet drugs, had a body mass index (BMI) of > 40 kg/m^2^, or refused to participate in the study.

Prior to TKA, patients were randomly assigned 1:1 to a group that received a tourniquet during TKA or to a group that did not. Random numbers were generated using a computer algorithm and sealed in opaque envelopes. Each patient was asked to select an envelope, inside which their group allocation was indicated. Observers who collected data after surgery were not involved in the surgery and were unaware of the group allocation.

### Anesthesia and surgery

All patients in our study received general anesthesia involving induction with midazolam (0.02–0.03 mg/kg), propofol (1–2 mg/kg), sulfentanyl (0.3–0.5 μg/kg), and rocuronium (0.6–1.0 mg/kg), which were delivered by intravenous bolus injection. Exceptions were patients older than 60 years, who did not receive midazolam. Anesthesia was maintained through continuous intravenous infusion of remifentanil (0.1–0.2 μg/kg·min) and continuous inhalation of sevoflurane. Rocuronium was added every 40–60 min at 25–33% of the induction dose. Sulfentanyl (5ug) was added every hour.

Intraoperative blood pressure was recorded every 3 min using an electrocardiogram and an upper-arm sphygmomanometer. Intraoperative blood pressure was maintained at baseline in the tourniquet group, or at approximately 70% of baseline in the non-tourniquet group. The target blood pressure was achieved through intravenous injection of m-hydroxylamine, ephedrine, and nicardipine.

All patients received antibiotics at 0.5–2 h before surgery. At 10 min before surgery, all patients received intravenous dexamethasone (10 mg) and intravenous tranexamic acid (60 mg/kg). Previous studies have confirmed the efficacy and safety of a preoperative high-dose (60 mg/kg) combined with postoperative multiple-dose tranexamic acid sequential application regimen [27–32]. Immediately before surgery, a tourniquet was applied at the base of the thigh in the tourniquet group and inflated to 100 mmHg above baseline systolic pressure. All surgeries were conducted by the same team of surgeons at our hospital, who had more than 10 years of experience in total joint arthroplasty, and were performed using a standardized medial parapatellar approach. All patients received the same type of cemented posterior-stabilized prosthesis (DePuy Synthes, Johnson and Johnson, New Brunswick, USA). During surgery, intramedullary guides were used for femoral preparation and extramedullary guides for tibial preparation. No postoperative drain was used.

### Postoperative management

All patients stopped using antibiotics within 24 h after surgery, and all received intravenous tranexamic acid (1 g) at 3, 6, 12, and 24 h after surgery. All patients received intravenous dexamethasone (10 mg) on postoperative day 1 and intravenous dexamethasone (5 mg) on postoperative day 2, and they began to receive oral prednisone (10 mg) from postoperative day 2 onward in order to control pain and reduce inflammation. All patients were required to start functional exercise as soon as they had recovered from anesthesia and to begin walking under pain control from postoperative day 1. Every patient received a lower-extremity pump and a subcutaneous injection of low-molecular-weight heparin (2000 IU) to prevent deep vein thrombosis from postoperative day 1. The patients were discharged on the third day after the operation if they had no signs of complications and could walk independently.

All patients underwent Doppler ultrasonography either immediately if they showed any sign of deep vein thrombosis or otherwise on postoperative day 2 or 3 and 14. Patients were scheduled for computed tomography angiography if they suddenly experienced chest discomfort or breathing difficulties, if they coughed up pink foamy sputum, or if they exhibited other symptoms suggestive of pulmonary embolism.

Blood transfusion was performed according to the guidelines of the Chinese Ministry of Health [33]: transfusions were given to patients whose hemoglobin level was lower than 70 g/L and who did not present clinical symptoms or to those whose hemoglobin level was lower than 100 g/L and who presented anemia-related organ dysfunction, intolerable anemia symptoms, or ongoing hidden blood loss. Albumin was used for patients whose albumin level was less than 35 g/L for 2 consecutive days after surgery. Twenty grams of albumin were used each time and albumin levels were rechecked. Whether to use additional albumin was determined according to the results of the re-examination.

### Data extraction and outcomes

Two investigators independently collected the following information from each patient: (1) basic information such as age, sex, height, weight, BMI, and comorbidities; (2) postoperative length of stay and overall length of hospitalization; (3) perioperative laboratory values, including pre- and postoperative hematocrit (Hct), hemoglobin (Hb), C-reactive protein (CRP), interleukin-6 (IL-6), fibrin degradation product (FDP), and D-dimer; (4) perioperative range of knee motion (ROM), knee circumference, and knee swelling rate; (5) intraoperative systolic blood pressure and blood loss; (6) postoperative VAS pain score; and (7) complications.

The primary outcome was perioperative blood loss, comprising total blood loss (TBL), intraoperative blood loss (IBL), and hidden blood loss (HBL). TBL was calculated using the Gross formula [34]:

$${\text{TBL}} = {{{\text{PBV}} \times ({\text{Hct}}_{{{\text{pre}}}} \, - \,\,{\text{Hct}}_{{{\text{post}}}} )} \mathord{\left/ {\vphantom {{{\text{PBV}} \times ({\text{Hct}}_{{{\text{pre}}}} \, - \,\,{\text{Hct}}_{{{\text{post}}}} )} {{\text{Hct}}_{{{\text{avg}}}} }}} \right. \kern-\nulldelimiterspace} {{\text{Hct}}_{{{\text{avg}}}} }},$$

where PBV is the predictive blood volume; Hct_pre_ is the preoperative Hct level; Hct_post_ is the lowest postoperative Hct level, which usually occurred on postoperative day 2 or 3; and Hct_avg_ is the average of Hct_pre_ and Hct_post_. PBV was calculated using the formula [35]

$${\text{PBV = [k}}_{1} \times {\text{height (m)}}^{3} ] + [{\text{k}}_{2} \times {\text{weight (kg)] + k3,}}$$

where *k*_1_ = 0.3669, *k*_2_ = 0.03219, *k*_3_ = 0.6041 for men, or *k*_1_ = 0.3561, *k*_2_ = 0.03308, and *k*_3_ = 0.1833 for women. HBL was defined as the difference between TBL and IBL.

Secondary outcomes were surgery duration, postoperative laboratory indices of inflammation and fibrinolysis, range of knee motion, VAS pain score, knee circumference, knee swelling rate, homologous transfusion, albumin use, and complications. Complications included postoperative hypertension, deep vein thrombosis, pulmonary embolism, calf muscular venous thrombosis, aseptic or septic wound complications, periprosthetic joint infection, 30-day mortality, and 90-day readmission. Postoperative hypertension was defined as a systolic pressure  of > 160 mmHg within 2 h after surgery as determined by electrocardiography and an upper-arm sphygmomanometer in the ward [36]. Systolic pressure was also recorded postoperatively using electrocardiography in the ward. Wound complications were defined as the need for intervention, such as superficial surgical debridement, re-suture, or a longer hospital stay [37]. If the wound showed secretion, at least two samples were cultured to test for the presence of bacteria. If two cultures were positive for homogeneous bacteria, wound complications were classified as septic [37]. Otherwise, wound complications were classified as aseptic [37]. The knee circumference was measured at the thigh at a position 10 cm above the upper edge of the patella when the patient was in a supine position with the knee straight. Knee swelling rate was calculated using the formula

$${\text{Knee swelling rate = }}{{{\text{(}}C_{{{\text{post}}}} \, - \,C_{{{\text{pre}}}} )} \mathord{\left/ {\vphantom {{{\text{(}}C_{{{\text{post}}}} \, - \,C_{{{\text{pre}}}} )} C}} \right. \kern-\nulldelimiterspace} C}_{{{\text{pre}}}} ,$$

where *C*_pre_ is the preoperative knee circumference and *C*_post_ is the postoperative knee circumference measured on postoperative days 1, 2, and 3.

### Statistical analysis

The minimum sample size was estimated based on a previous study in our institute [38]. In previous studies, the average duration of surgery without a tourniquet was 84.9 min with a standard deviation of 20.1 min. We assume that 10 min is the least clinically significant reduction in duration of surgery due to tourniquet application. The test power (1 −* β*) was 0.8 and the alpha error rate was 0.05. The lost-to-follow-up rate was set at 0.05. Calculations indicated that at least 68 patients were required for each group.

Statistical analysis was performed using SPSS 22.0 (IBM, Armonk, NY, USA). Continuous data with a normal distribution were expressed as mean ± standard deviation (SD), while categorical data were expressed as frequencies. Inter-group differences were analyzed for significance using the Mann–Whitney *U* test in the case of continuous data that were skewed or showed unequal variance, or using the independent samples *t*-test in the case of normally distributed continuous data. Inter-group differences in categorical data were assessed using the chi-squared test or Fisher’s exact test as appropriate. Differences with *P* < 0.05 were considered significant.

## Results

**Fig. 1:**
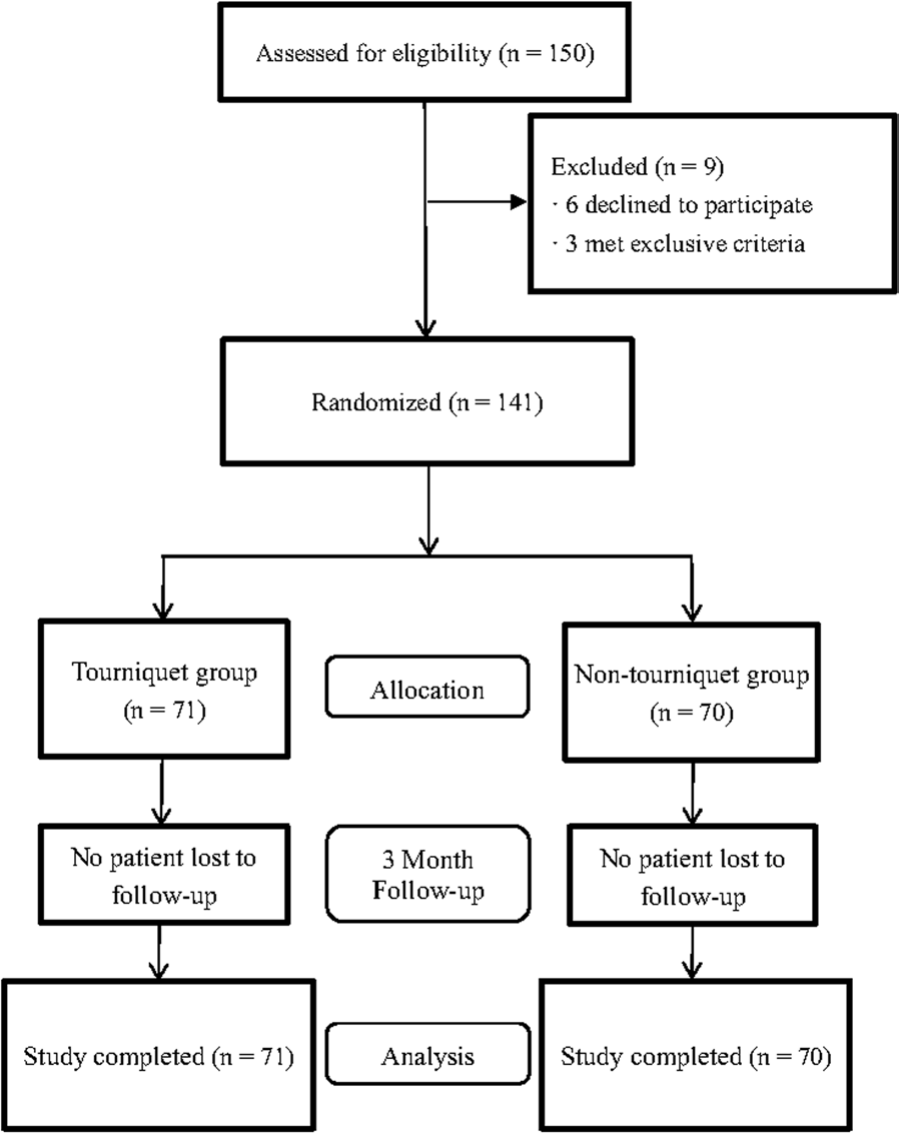
Flowchart of patient enrollment

In this study, 150 patients were candidates for cemented TKA at our hospital. Six patients refused to participate in the study. Two patients were excluded due to coagulopathy and 1 patient was excluded due to severe anemia. We finally enrolled 141 patients in our study, allocating 71 patients to the tourniquet group and 70 to the non-tourniquet group (Fig. 1). All patients were followed up for at least 3 months. The two groups did not differ significantly in age, sex distribution, comorbidities, ROM, knee circumference, or preoperative values for Hb, Hct, CRP, IL-6, FDP, or D-dimer (Table 1). Similarly, they did not differ significantly in postoperative stay or overall hospital stay.

**Table 1:** Baseline characteristics of and comorbidities in patients undergoing TKA with or without a tourniquet

Characteristic	No tourniquet (*n* = 70)	Tourniquet (*n* = 71)	P*
Mean age, years	66.0 ± 8.6	66.3 ± 8.4	0.805
Female	57 (81.4)	60 (84.5)	0.627
BMI, kg/m^2^	26.7 ± 4.1	26.4 ± 3.5	0.629
Length of hospital stay, days	5.4 ± 1.1	5.1 ± 0.4	0.109
Postoperative length of stay, days	3.3 ± 1.0	3.1 ± 0.3	0.074
Comorbidities			
Hypertension	27 (38.6)	20 (28.2)	0.190
Diabetes	8 (11.2)	4 (5.6)	0.218
Coronary heart disease	2 (2.9)	3 (4.2)	0.661
Hypothyroidism	3 (4.3)	1 (1.4)	0.304
COPD	2 (2.9)	2 (2.8)	0.989
Chronic liver disease	1 (1.4)	2 (2.8)	0.568
Renal insufficiency	3 (4.3)	0	0.078
Anemia	2 (2.9)	1 (1.4)	0.551
Autoimmune disease	4 (5.7)	5 (7.0)	0.747
Preoperative laboratory values			
Preoperative hemoglobin, g/L	133.2 ± 10.7	135.1 ± 11.5	0.327
Preoperative hematocrit, %	40.9 ± 3.2	41.5 ± 3.3	0.324
Preoperative CRP, mg/L	3.83 ± 2.04	3.23 ± 1.88	0.088
Preoperative IL-6, pg/mL	4.24 ± 3.97	3.51 ± 5.69	0.381
Preoperative FDP, mg/L	2.88 ± 1.23	2.79 ± 1.09	0.654
Preoperative D-dimer, mg/L	0.58 ± 0.43	0.49 ± 0.31	0.169
Preoperative systolic pressure, mmHg	136.7 ± 15.3	134.8 ± 15.5	0.459
Preoperative ROM, degrees	97.8 ± 15.0	98.1 ± 11.5	0.915
Preoperative knee circumference, cm	39.6 ± 1.5	40.2 ± 1.7	0.117

Values are *n* (%) or mean ± SD

* Based on Levene’s test or the chi-squared test

**Fig. 2:**
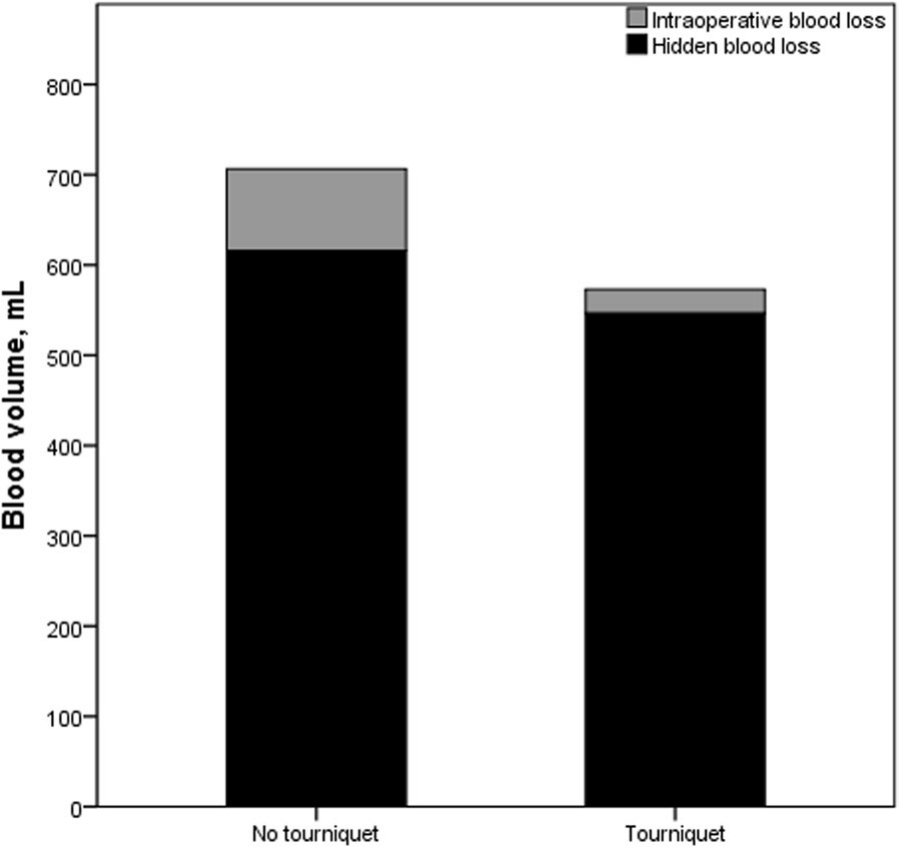
Comparison of hidden blood loss (*black*) and intraoperative blood loss (*gray*) between the two groups

The use of a tourniquet was associated with significantly higher intraoperative systolic pressure and a shorter surgery (Table 2). It was also associated with significantly lower IBL and TBL (Fig. 2). In contrast, the use of a tourniquet did not significantly affect HBL, postoperative ROM, or the levels of CRP, IL-6, FDP, or D-dimer on postoperative days 1, 2, 3, or 14 (Table 3). Moreover, the use of a tourniquet did not significantly increase the postoperative VAS pain score, knee circumference, or knee swelling rate (Table 4).

**Table 2:** Comparison of blood loss, intraoperative systolic pressure, operating time, and postoperative ROM between the two groups

Outcome	No tourniquet (*n* = 70)	Tourniquet (*n* = 71)	P*
Intraoperative systolic pressure, mmHg	110.3 ± 9.0	118.2 ± 10.7	< 0.001*
IBL, mL	90.4 ± 22.0	26.8 ± 13.9	< 0.001*
TBL, mL	706.4 ± 336.1	573.0 ± 228.4	0.007*
HBL, mL	616.0 ± 339.6	546.2 ± 228.0	0.155
Duration of surgery, min	77.3 ± 15.5	65.2 ± 10.7	< 0.001*
Postoperative ROM**, degrees	108.0 ± 8.3	110.0 ± 7.4	0.137

Data are shown as mean ± SD unless otherwise noted

* Based on Levene’s test

** On postoperative day 3

**Table 3:** Comparison of postoperative indices of inflammation and fibrinolysis between the two groups (Figs. 3)

Value	No tourniquet (*n* = 70)	Tourniquet (*n* = 71)	P*
Postoperative CRP, mg/L			
Postop day 1	15.21 ± 7.67	15.27 ± 9.36	0.973
Postop day 2	51.60 ± 35.85	44.09 ± 38.06	0.237
Postop day 3	66.04 ± 24.07	72.54 ± 27.86	0.475
Postop day 14	6.26 ± 5.41	7.00 ± 5.04	0.454
Postoperative IL-6, pg/mL			
Postop day 1	24.73 ± 17.67	27.61 ± 21.27	0.399
Postop day 2	29.15 ± 13.93	25.44 ± 12.13	0.130
Postop day 3	20.36 ± 8.71	18.34 ± 15.06	0.602
Post day 14	4.70 ± 2.60	5.80 ± 3.66	0.074
Postoperative FDP, mg/L			
Postop day 1	5.39 ± 2.60	5.34 ± 2.91	0.927
Postop day 2	4.91 ± 1.77	4.14 ± 1.16	0.114
Postop day 3	5.24 ± 1.74	4.66 ± 2.33	0.478
Post day 14	7.97 ± 2.37	8.88 ± 2.67	0.109
Postoperative D-dimer, mg/L			
Postop day 1	2.37 ± 1.48	2.31 ± 1.85	0.834
Postop day 2	1.22 ± 0.18	1.10 ± 0.15	0.093
Postop day 3	2.36 ± 1.00	1.92 ± 1.15	0.312
Postop day 14	4.39 ± 2.30	5.16 ± 2.30	0.103

Data are shown as mean ± SD unless otherwise noted

* Based on Levene’s test

**Table 4:** Comparison of postoperative VAS pain score, knee circumference, and thigh swelling rate

Value	No tourniquet (*n* = 70)	Tourniquet (*n* = 71)	P *
Postoperative VAS pain score			
Postop day 1	4.0 ± 0.7	3.8 ± 1.0	0.513
Postop day 2	3.6 ± 0.8	3.6 ± 0.7	0.884
Postop day 3	3.0 ± 0.8	2.8 ± 0.8	0.211
Postoperative knee circumference, cm			
Postop day 1	41.5 ± 1.8	41.7 ± 2.3	0.649
Postop day 2	41.4 ± 1.6	41.7 ± 1.4	0.339
Postop day 3	41.1 ± 1.2	41.0 ± 1.0	0.613
Knee swelling rate, %			
Postop day 1	4.98 ± 5.96	3.52 ± 8.05	0.359
Postop day 2	4.59 ± 5.86	3.36 ± 5.55	0.336
Postop day 3	3.75 ± 2.65	2.60 ± 2.38	0.062

Data are shown as mean ± SD unless otherwise noted

* Based on Levene’s test

**Fig. 3:**
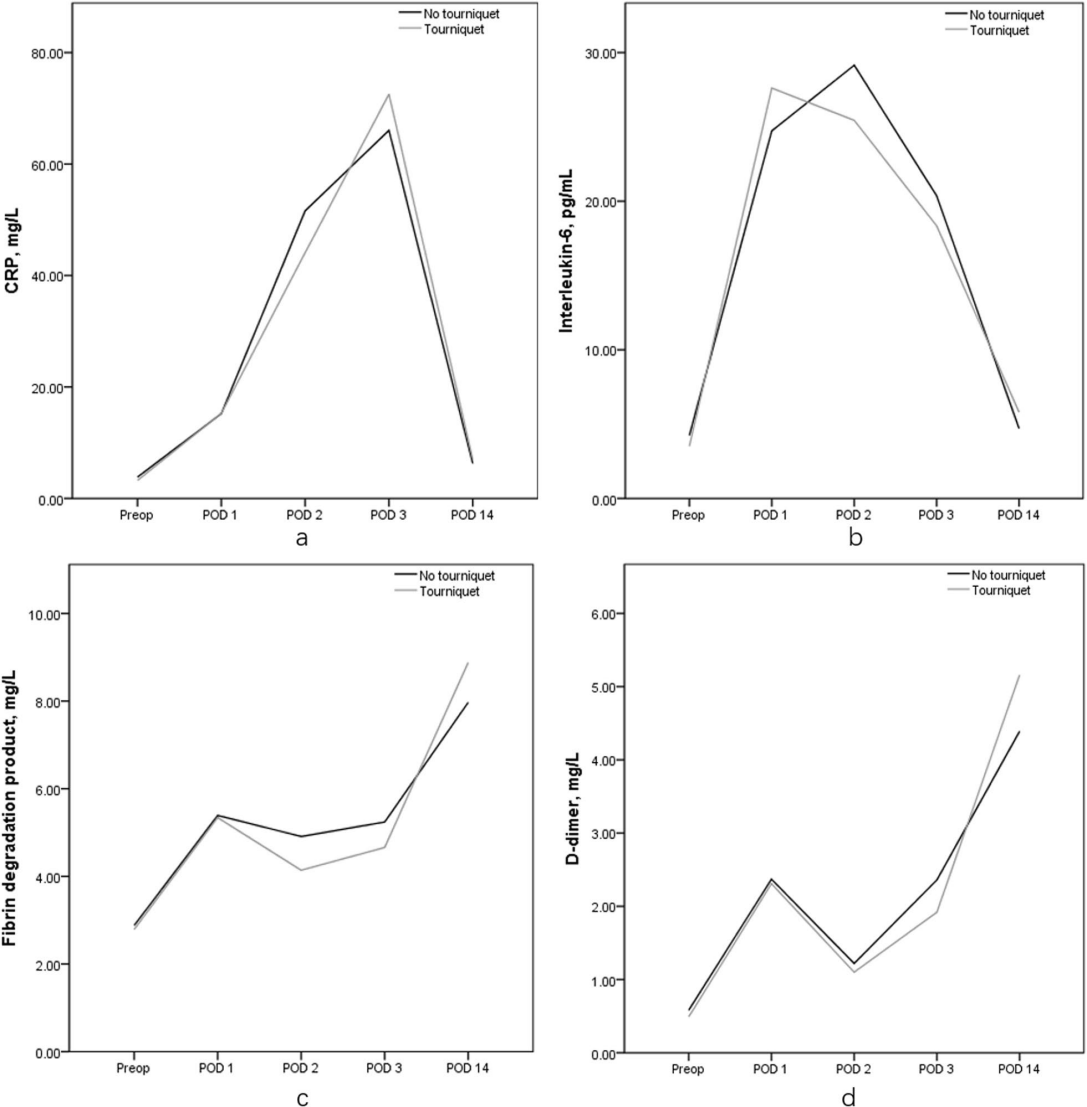
**a** CRP levels in the two groups at different time points. **b** IL-6 levels in the two groups at different time points.** c** FDP levels in the two groups at different time points.** d** D-dimer levels in the two groups at different time points

In both groups, CRP levels peaked on postoperative day 3, while IL-6 levels peaked on postoperative day 2. Levels of both FDP and D-dimer peaked on postoperative day 14 (Fig. 3).

The two groups did not show significant differences in postoperative hypertension, deep vein thrombosis, pulmonary embolism, calf muscular venous thrombosis, septic or aseptic wound complications, 90-day readmission, or albumin use (Table 5). No patients died within 30 days, had a periprosthetic joint infection, or required homologous transfusion during follow-up.

**Table 5:** Comparison of complications, homologous transfusions, and albumin use between the two groups

Outcome	No tourniquet (*n* = 70)	Tourniquet (*n* = 71)	P*
Postoperative hypertension	9 (12.9)	12 (14.3)	0.500
Deep vein thrombosis	1 (1.4)	0	0.312
Pulmonary embolism	1 (1.4)	0	0.312
Calf muscular venous thrombosis	2 (2.9)	2 (2.8)	0.989
Periprosthetic joint infection	0	0	NA
Wound complications			
Septic	1 (1.4)	0	0.312
Aseptic	0	0	NA
30-day mortality	0	0	NA
90-day readmission	2 (2.9)	0	0.151
Homologous transfusion	0	0	NA
Albumin use	10 (14.3)	6 (8.5)	0.275

Data are shown as *n* (%) unless otherwise noted

*NA* not applicable

* Based on the chi-squared test

## Discussions

The most important finding of our study was that applying a tourniquet to patients undergoing cemented TKA involving multiple doses of dexamethasone and tranexamic acid reduced IBL without increasing HBL or the risk of complications. It also significantly shortened surgery and reduced intraoperative controlled hypotension requirements.

Several studies have shown that tourniquets can reduce IBL and thereby help ensure a bloodless surgical field in orthopedic procedures such as TKA [39–41]. Indeed, the bloodless surgical field may reduce the time needed to ensure hemostasis and identify structures during the procedure, which may help explain why the tourniquet shortened surgery in our study. The concepts of minimally invasive surgery and fine operation are related to careful hemostasis. Intraoperative trauma was minimized and an unnecessary release and synovectomy were not performed. For patients who use a tourniquet, every wound bleeding point should be completely electrocoagulated to stop the bleeding. Otherwise, obvious bleeding may occur after the tourniquet is loosened. Tourniquets have also been shown to significantly reduce TBL [42, 43]. Our results are consistent with that literature. Reducing IBL in TKA has been reported to improve cement penetration and initial fixation strength, reducing the long-term risk of aseptic loosening [3]. Whether tourniquets affect this is unclear, and we could not address this question because of the relatively short follow-up.

In addition, we found that tourniquet use did not increase HBL, in contrast to previous work [2, 5, 7]. The increase in HBL due to tourniquets has been attributed to the exacerbation of hyperfibrinolysis caused by surgical trauma, which could lead to knee swelling and pain [23, 44]. Thus, the key to decreasing the TBL due to tourniquets is to inhibit postoperative hyperfibrinolysis. Tranexamic acid is an anti-fibrinolytic agent that has been proven to inhibit plasminogen activation and reduce hidden blood loss [13, 18–23]. Using a tourniquet with intravenous tranexamic acid did not increase the levels of FDP and D-dimer after surgery, nor did they significantly increase hidden blood loss, knee swelling, or pain.

Like a tourniquet, hypotension anesthesia during TKA has been reported to provide a clear surgical field as well as reduce HBL and the risk of deep vein thrombosis [45–47]. On the other hand, it may increase the risk of postoperative acute kidney injury and myocardial damage, with the severity of damage being proportional to the extent and duration of hypotension [48, 49]. Applying a tourniquet may avoid these problems because, as we found here, it was associated with higher intraoperative systolic blood pressure. Another advantage of tourniquets is that they are more straightforward and less demanding to apply than hypotension anesthesia.

Applying tourniquets during TKA has been shown to increase the levels of the inflammatory factors CRP and IL-6, probably as a result of ischemia and damage to soft tissue [5]. Furthermore, the inflammatory response after TKA causes severe pain and postoperative nausea and vomiting, leading to delayed early rehabilitation and hospital discharge [9–11]. However, we found that tourniquets did not significantly alter the changes in the levels of CRP or IL-6 during TKA, or the VAS pain score or knee diameter. This may reflect that all patients received multiple doses of dexamethasone, which can effectively inhibit postoperative inflammatory responses, as reported by Xu et al. [9]. Tourniquet use combined with perioperative dexamethasone can reduce tourniquet-related inflammatory responses, promote postoperative recovery, and shorten the postoperative length of stay.

Xie et al. have reported that using a tourniquet could increase the incidence of postoperative deep vein thrombosis [42]. Zhou et al. demonstrated that high pressure and prolonged ligation lead to limb ischemia, and when the tourniquet is relieved, ischemia–reperfusion leads to secondary endothelial injury and thrombus formation [50]. Using tourniquets causes lower-limb ischemia and releases inflammatory cytokines to promote thrombus formation [51]. Our insistence on early ambulation after TKA and suppressing inflammation with multiple doses of dexamethasone may have also helped reduce the risk of thrombosis.

Our results should be verified and extended in studies with a longer follow-up in order to detect delayed or long-term complications. Future work with a longer follow-up should examine the safety and efficacy of tourniquets for patients undergoing cemented TKA involving multiple doses of dexamethasone and tranexamic acid, as well as the effects of tourniquets on the speed and extent of joint function recovery, based on the repeated measurement of the postoperative ROM. Ultimately, our findings should be validated with large multicenter studies.

## Conclusions

Our randomized trial suggests that applying tourniquets to patients undergoing TKA involving multiple doses of dexamethasone and tranexamic acid can reduce perioperative blood loss without increasing HBL, postoperative inflammation, or risk of thrombosis, even though these have been described as adverse effects of tourniquets in orthopedic procedures such as TKA.

## Data Availability

The datasets used and/or analyzed during the current study are available from the corresponding author on reasonable request.

## Abbreviations

TKA: Total knee arthroplasty
VAS: Visual analog scale
BMI: Body mass index
Hct: Hematocrit
Hb: Hemoglobin
CRP: C-reactive protein
IL-6: Interleukin-6
FDP: Fibrin degradation product
ROM: Range of knee motion
TBL: Total blood loss
IBL: Intraoperative blood loss
HBL: Hidden blood loss
COPD: Chronic obstructive pulmonary disease
